# Interleukin-36 Receptor Signaling Attenuates Epithelial Wound Healing in C57BL/6 Mouse Corneas

**DOI:** 10.3390/cells12121587

**Published:** 2023-06-08

**Authors:** Qi Chen, Nan Gao, Fu-Shin Yu

**Affiliations:** Departments of Ophthalmology, Visual and Anatomical Sciences, School of Medicine, Wayne State University, Detroit, MI 48201, USAec0422@wayne.edu (N.G.)

**Keywords:** wound healing, diabetic keratopathy, interleukin-36

## Abstract

The IL-36 cytokines are known to play various roles in mediating the immune and inflammatory response to tissue injury in a context-dependent manner. This study investigated the role of IL-36R signaling in mediating epithelial wound healing in normal (NL) and diabetic (DM) C57BL/6 mouse corneas. The rate of epithelial wound closure was significantly accelerated in IL-36 receptor-deficient (IL-36R^−/−^) compared to wild-type (WT) mice. Wounding increased IL-36α and -36γ but repressed IL-36R antagonist (IL-36Ra) expression in B6 mouse corneal epithelial cells. The wound-induced proinflammatory cytokines CXCL1 and CXCL2 were dampened, while the antimicrobial peptides (AMPs) S100A8 and A9 were augmented in IL-36R^−/−^ mouse corneas. Intriguingly, the expression of AMP LCN2 was augmented at the mRNA level. LCN2 deficiency resulted in an acceleration of epithelial wound healing. IL-36R deficiency also greatly increased the healing rate of the corneal epithelial wound in DM mice. IL-36R deficiency also suppressed IL-1β, IL-1Ra, and ICAM expression in unwounded-DM mice and wounded NL corneas. Opposing IL-1β and ICAM, the expression of IL-Ra in DM corneas of IL-36R^−/−^ mice was augmented. The presence of recombinant IL-1Ra and IL-36Ra accelerated epithelial wound closure in T1DM corneas of B6 mice. Our study revealed an unprecedented role of IL-36R signaling in controlling corneal epithelial wound healing in normal (NL) and diabetic (DM) mice. Our data suggest that IL-36Ra, similar to IL-1Ra, might be a therapeutic reagent for improving wound healing and reducing wound-associated ulceration, particularly in the cornea and potentially in the skin of DM patients.

## 1. Introduction

The role of IL-36R signaling has been shown to have both proinflammatory effects and a potential protective role [1,2,3,4]. Mice deficient in IL-36R exhibited defective recovery following dextran sodium sulfate-induced damage and impaired closure of colonic mucosal biopsy wounds. This coincided with impaired neutrophil accumulation in the wound bed [5]. In the inflammatory bowel disease model, mucosal damage activated IL-36R(+) colonic fibroblasts via Myd88 to induce the expression of GM-CSF and IL-6. Defective IL-36R signaling causes high susceptibility to acute dextran sodium sulfate colitis and impairs wound healing [6]. Moreover, IL-36γ induces the expression of REG3A via the activation of the TLR3–SLUG–VDR axis. REG3A regulates keratinocyte proliferation and differentiation, thus promoting wound re-epithelialization and wound healing [7]. On the other hand, loss-of-function homozygous or compound heterozygous mutations in IL36RN have been implicated in the pathogenesis of various skin disorders [8,9]. Deficient or uncontrolled IL-36R signaling resulted in delayed healing of full-thickness excisional skin wounds due to excessive recruitment of immune cells, neutrophils, and macrophages. It also increased the expression of cytokines such as IL-36γ, CXCL1, and TGF-β [9]. *IL-36Ra* deficiency exacerbated cutaneous I/R injury due to excessive inflammatory cell recruitment, NET formation, and excessive cytokine and chemokine production via the TLR4 pathway by HMGB1 released from epidermal apoptotic cells [10]. However, the roles of IL-36R signaling in corneal epithelial wound healing and hyperglycemia-impaired epithelial wound closure remain undetermined.

IL-36 cytokines are the newest members of the IL-1 superfamily. As IL-1 cytokines, they have been shown to play roles in tissue homeostasis and inflammation [11,12]. There are three agonists, IL-36α, IL-36β, and IL-36γ, which share a common heterodimeric receptor, IL-36R [13]. The binding of IL-36 cytokines to IL36R recruits IL-1RAcP, leading to activation of the NF-kb and MAPK pathways and inducing downstream gene transcription [14]. IL-36/36R signaling is activated in response to bacterial, viral, and mycobacterium infection, resulting in the elevation of inflammatory and antimicrobial activities [15,16,17]. Notably, IL-38 was found to attenuate sepsis by decreasing inflammation and increasing bacterial clearance, suggesting that modulation of IL-36/IL-36R activity might be utilized to control or treat bacterial infection [18]. In mouse models of tissue infection, IL-36 agonists exhibited both beneficial and detrimental roles in a tissue- and pathogen-specific manner [15,17,19]. IL-36 acts synergistically with TLRs to induce the expression of antimicrobial proteins (AMPs), such as cathelicidin (LL37) and lipocalin2 (LCN2), which is a potent neutrophil chemoattractant and participates in psoriasis [20]. Using siRNA silencing, we recently demonstrated that IL-36Ra and IL-1Ra have opposing roles in the innate immune response to *Pseudomonas aeruginosa* infection [21]. This suggests that the IL-36/IL36R axis may antagonize the IL-1/IL-1R pathway [21]. However, knowledge of the role of IL-36R signaling in corneal wounding in NL and DM mice is limited.

Diabetes mellitus (DM) is a major disease worldwide, and its prevalence has risen significantly in the past few decades. Although the major ocular complication is diabetic retinopathy, corneal diseases, such as diabetic neurotrophic keratopathy (DNK) or diabetic kerato/epitheliopathy [22,23], can not only develop but are also difficult to manage [24,25,26]. DNK is a component of diabetic peripheral neuropathy (DPN) and a major cause of the morbidity of the cornea [24]. Hyperglycemia also causes changes in the basement membrane and delayed epithelial wound healing, which may increase the opportunity for microbial infection of the corneas [27]. The CDC recently, as of 15 May, reported 4 deaths, 14 patients with vision loss, and an additional 4 patients with enucleation, following infection with the carbapenem-resistant *Pseudomonas aeruginosa* strain, linked to using EzriCare Artificial Tears, highlighting the importance for understanding bacterial keratitis in diabetic patients who are more likely to use Artificial Tears. In this study, we investigated the role of IL-36R signaling in mediating epithelial wound healing in NL and DM corneas using *IL-36R^−/−^* and *LCN2^−/−^* mice. We found that *IL-36R* deficiency increased the rate of epithelial wound healing and augmented wound-induced AMP LCN2 expression. Furthermore, we observed that *IL-36R* deficiency and IL-36Ra accelerated delayed epithelial wound healing in DM corneas. This suggests that IL-36Ra may have potential use in treating impaired wound healing and ulceration in DM corneas.

## 2. Material and Methods

### 2.1. Animals

Age- and sex-matched C57BL/6 WT mice were purchased from The Jackson Laboratory (Bar Harbor, ME, USA). Breeding pairs of *IL-36R^−/−^*, *IL-36α^−/−^*, and *IL-36γ^−/−^* mice with a C57BL/6 genetic background were provided by DrBy Dr. Theodore Standiford of University of Michigan [28]. *LCN2^−/−^* mice were purchased from The Jackson Laboratory (Bar Harbor, ME, USA).

### 2.2. Animals and Induction of Diabetes

All experimental and animal care protocols were approved by the IACUC of Wayne State University. All investigations conformed to the guidelines of the Association for Research in Vision and Ophthalmology (ARVO) Statement on the Use of Animals in Ophthalmic and Vision Research, the NIH, and the Animal Investigation Committee of Wayne State University. Six-week-old C57BL/6 mice (males or females) were purchased from Jackson Laboratory and induced to develop diabetes with STZ (Sigma, St. Louis, MO, USA), as described previously [29,30]. Mice were considered diabetic with blood glucose levels > 350 mg/dL within 8 weeks postinjection and thereafter. 

### 2.3. Corneal Epithelial Debridement Wounds

Diabetic and age-matched normal mice were anesthetized by an intraperitoneal injection of 7 mg/kg xylazine and 70 mg/kg ketamine, and a 2 mm circular wound was first demarcated with a trephine in the cornea, followed by the removal of corneal epithelial cells (CECs) within the circle using a blunt scalpel blade under a Zeiss dissecting microscope. Two corneas were pooled in a tube and stored at −80 °C. Cells collected during epithelium debridement were marked as unwounded (0 h). The progress of wound healing was monitored by fluorescence staining and photographed with a slit lamp microscope. At the end of healing, the corneas were either snap frozen in OCT Compound (Torrance, CA, USA) or marked with the same size trephine for CEC collection.

### 2.4. Subconjunctival Injection of Proteins 

The mice were given subconjunctival injections with a volume of 5 µL per injection containing 150 ng recombinant protein IL36Ra (R&D systems, Minneapolis, MN, USA) or 150 ng anakinra (SOBI, Waltham, MA, USA) with PBS containing 0.1% bovine serum albumin as the control. 

### 2.5. PCR Analysis 

RNA was extracted from the collected CECs using an RNeasy Mini Kit (Qiagen, Valencia, CA, USA) and used for cDNA generation with an oligo (dT) primer, followed by analysis using real-time quantitative RT-PCR (qRT-PCR) with SYBR Green (StepOnePlus; Applied Biosystems, Carlsbad, CA, USA), with β-actin expression used as an internal control. qRT-PCR results were first normalized with the levels of β-actin and then compared with the levels of NL (value = 1) and presented as fold changes. 

### 2.6. Statistical Analysis 

The statistical analyses were performed with GraphPad Prism 6 software. Data are presented as means ± SDs. Experiments with two treatments and/or conditions were analyzed for statistical significance using a two-tailed Student’s *t*-test. A Bonferroni post-test was performed to determine statistically significant differences. Significance was accepted at *p* < 0.05. Experiments were repeated at least twice to ensure reproducibility.

## 3. Results

### 3.1. IL-36/IL-36R Signaling Plays a Detrimental Role in Corneal Epithelial Wound Closure in Normoglycemia B6 Mouse Corneas 

**Figure 1:**
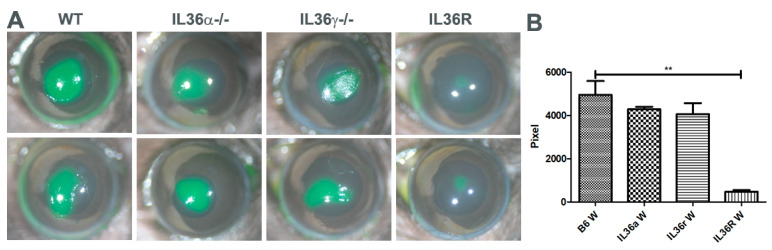
*IL-36R* deficiency promotes epithelial wound closure in IL-36 knockout mice. A 2 mm epithelial debridement wound was created and allowed to heal for 24 h in wild-type (WT) and IL-36 cytokine and receptor knockout corneas. The remaining wounds were photographed by a camera (10×) (**A**), and wound sizes were measured using Image J 1.47 (**B**). The results are presented as the mean ± SD. *p* values were analyzed with a one-way ANOVA, followed by a Bonferroni test. *n* = 5, ** *p* < 0.01. The results are representative of three independent experiments.

Our previous study showed that IL-36/IL-36R signaling opposes the role of IL-1β/IL-1R in mediating corneal immune defense against Pseudomonas aeruginosa infection [21]. To investigate the role of IL-36R signaling in mediating corneal epithelial wound healing, we created 2 mm epithelial debridement wounds in WT, *IL-36α*, *IL-36γ*, and *IL-36R*-deficient mice and allowed the wounds to heal in vivo. Epithelial debridement was visualized by fluorescence staining at 0 and 24 h postwounding. As shown in Figure 1, compared to the wild-type mice (WT), *IL-36α^−/−^*, and *IL-36γ^−/−^* mice, *IL-36R* deficiency resulted in accelerated corneal wound healing at 24 h postwounding. However, there were no significant differences among WT, *IL-36α^−/−^*, and *IL-36γ^−/−^* mice.

### 3.2. Wound-Induced Expression of IL-36 Cytokines in B6 Mouse Corneas

**Figure 2:**
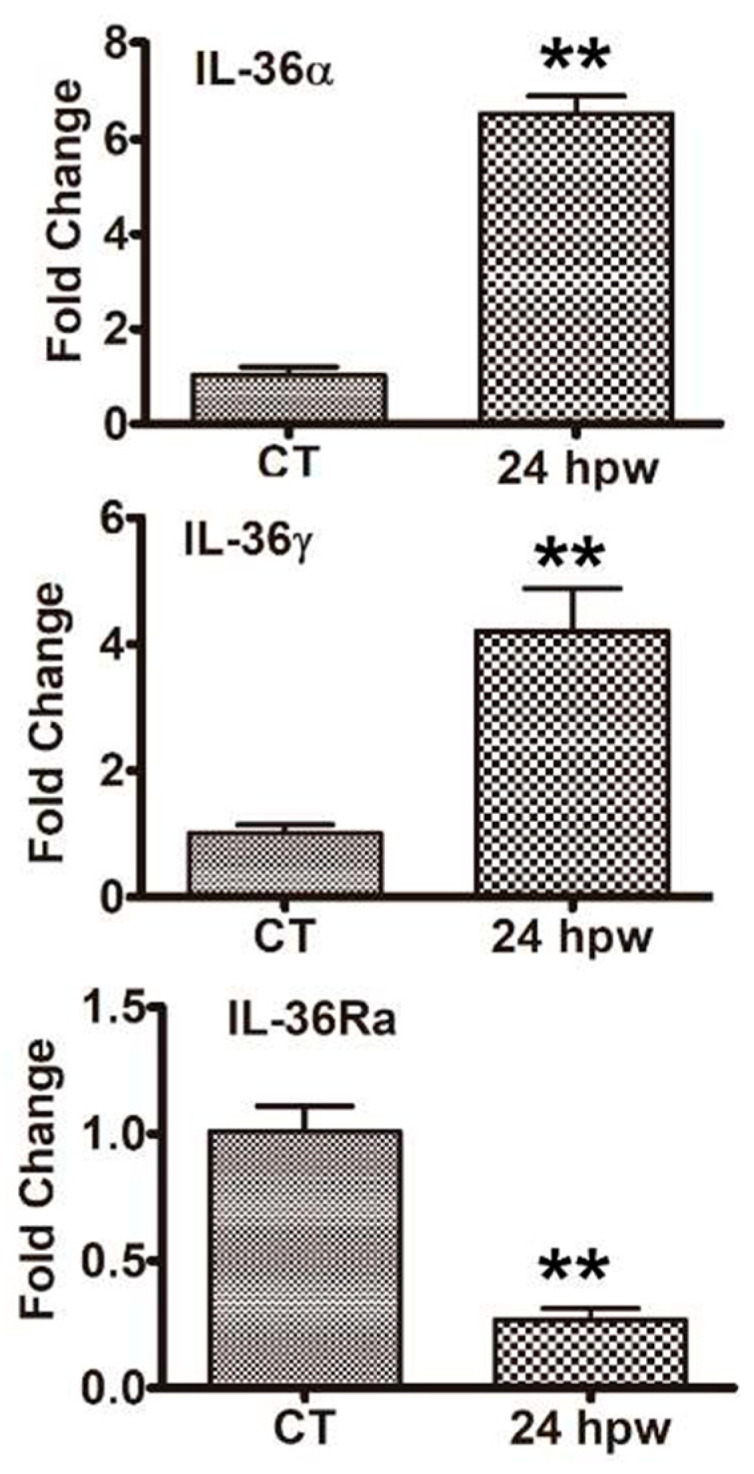
Wound induced the expression of *IL-36* Cytokines in B6 Mouse Corneas. A 2 mm epithelial debridement wound was created, and epithelial cells were collected as the control (CT). The wounds were allowed to heal for 24 h, and the epithelial cells that migrated into the original wound were scraped off and collected at 24 hpw. The collected epithelial cells were subjected to qPCR analyses of *IL-36α* cytokines, *-36γ*, and -*36rn*−−. The results are representative of two independent experiments (*n* = 3 each). ** *p* < 0.01, by two-tailed, unpaired Student’s *t* tests.

Our previous study revealed the upregulation of IL-36α and IL-36γ in response to microbial infection and a protective role of IL-36R signaling in infectious keratitis [2,21]. Using qPCR, we assessed the expression of IL-36 isoforms in corneal epithelial cells of B6 mice collected before wounding (the control) and at 24 h postwounding (hpw) (Figure 2). The levels of mRNA were increased by 6.36- and 4.13-fold for IL-36α and IL-36γ, respectively, while the expression of IL-36Ra was decreased by 3.77-fold in healing compared to control epithelial cells. 

### 3.3. Effects of IL36R Deficiency on the Expression of Cytokines and Antimicrobial Peptides in Wounded Corneas

**Figure 3:**
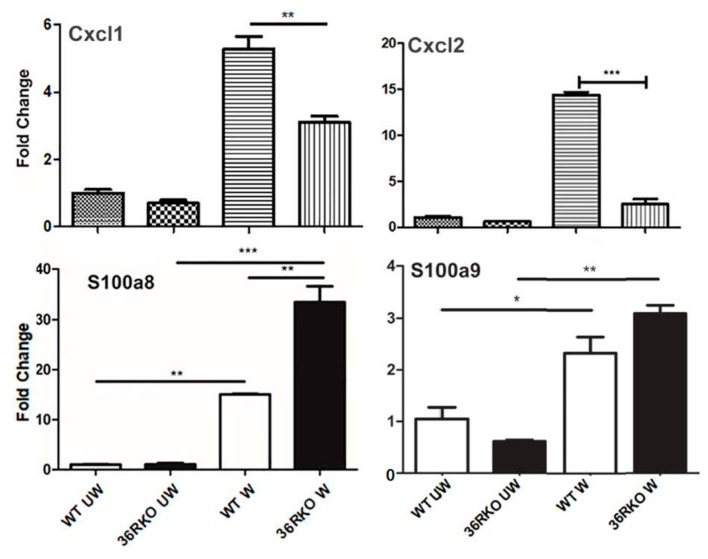
IL-36R plays a role in regulation of the expression of cytokines and antimicrobial peptides. Corneal epithelial cells collected from NL or DM B6 mice during wounding and at 24 hpw were subjected to real-time PCR analyses. Each sample was normalized with actin as the internal control, and the results are expressed as fold change compared with unwounded WT epithelial cells. The results are representative of two experiments, each with three samples derived from three mice. * *p* ≤ 0.05, ** *p* ≤ 0.01, *** *p* ≤ 0.001 by two-tailed, unpaired Student’s *t* tests.

To explore the underlying mechanism of how IL-36 signaling influences the outcome of epithelial wound closure, we used qPCR to assess the effects of *IL-36* deficiency on the expression of several genes that were previously shown by us to be involved in corneal innate defense and wound healing (Figure 3). The expression of *Cxcl1*, *Cxcl2* (human analogs of IL-8), *S100a8*, and *S100a9* (forming calprotectin) increased in response to wounding. The wound-induced increases in *Cxcl1* and *Cxcl2* at the mRNA level were dampened, while *S100a8* and *S100a9* were augmented in *IL-36R*-depleted B6 mouse corneas.

### 3.4. LCN2 Plays a Detrimental Role in Epithelial Wound Healing in B6 Mouse Corneas

**Figure 4:**
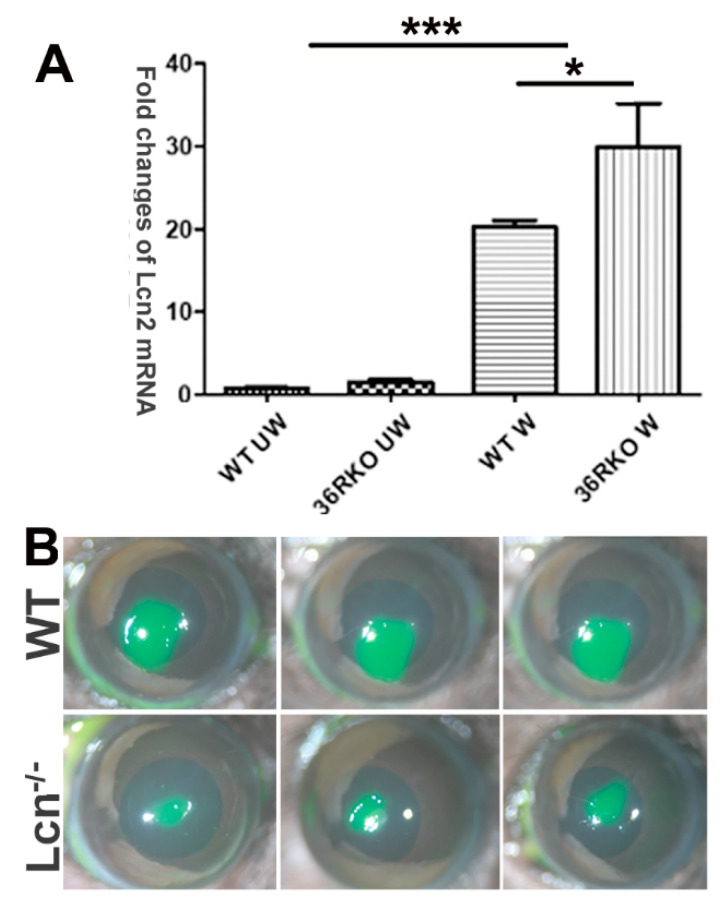
LCN2 plays a role in epithelial wound healing in B6 mouse corneas B6 mouse corneas were wounded as described in Figure 1. (**A**) Unwounded and healing corneal epithelial cells (24 hpw) of *WT* and *36R*-deficient mice were collected and processed for RT-PCR analysis of *LCN2*. The results were first normalized with β-actin levels and then compared with WT, unwounded cornea levels (value 1) and presented as the fold change (*n* = 3). * *p* < 0.05 and *** *p* < 0.001 by one-way ANOVA with Bonferroni’s post test. (**B**) *WT* and *LCN2* knockout mice were wounded, and the remaining wounds were photographed (10×) at 24 hpw. Three corneas are presented in panel (**B**).

LCN2 was shown to mitigate gut barrier injury by maintaining the homeostasis of the microbiota and exerting an antioxidant strategy, as well as by deactivating macrophages and inducing immune cell apoptosis to terminate systemic hyperinflammation [31]. However, its role in injury repair differs in a cell-content-dependent manner. In the skin, it promotes cell migration and wound healing [32] while playing an immunomodulatory role and having detrimental effects on spinal cord injury [33]. qPCR analysis revealed that *LCN2* expression was induced by wounding and that *IL36R* deficiency augmented this wound-induced upregulation (Figure 4A). To determine the role of LCN2 in corneal wound healing, we utilized LCN2 knockout mice and observed that *LCN2* deficiency markedly accelerated epithelial wound closure compared to that in wild-type B6 mice (Figure 4B). This suggests that LCN2, as a downstream effector of IL-36R signaling, plays a detrimental role in corneal wound healing.

### 3.5. IL-36R Deficiency Greatly Accelerates Epithelial Wound Closure in DM Corneas of B6 Mice 

The upregulation of IL-36 cytokines has been shown to be associated with various mouse models of kidney diseases, including lupus nephritis, diabetic nephropathy, traumatic kidney injury [27], and skin diseases such as psoriasis and atopic dermatitis [34]. To understand the role of IL-36R signaling in normal and diabetic corneal wound healing, we treated *IL-36R*-deficient mice (*IL-36^−/−^*) with streptozotocin to induce diabetes by administering multiple low doses of STZ (40 mg/kg, intraperitoneally) to mice on 5 consecutive days.

**Figure 5:**
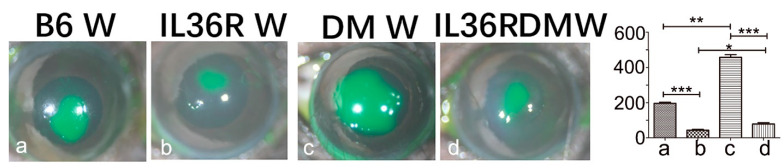
IL-36R deficiency promotes epithelial wound closure in *IL-36R* knockout mice. A 2 mm epithelial debridement wound was created in NL and DM corneas, and wounds were allowed to heal for 24 h in vivo. The remaining wounds were photographed (10×), and wound sizes were measured using Image J 1.47 (a, B6 W; b, IL36R W; c, DMW; d, IL36R DMW). The results are presented as the mean ± SD. *p* values were analyzed with a one-way ANOVA, followed by a Bonferroni test. *n* = 5, * *p* < 0.05, ** *p* < 0.01, *** *p* < 0.001. The results are representative of two independent experiments.

Figure 5 shows epithelial wound closure in NL and DM corneas. Compared to normoglycemia (B6 W), wound closure in hyperglycemia corneas (DM W) was delayed; the remaining wound sizes in DM corneas were 2.34-fold larger than those in NL corneas. Consistent with what is shown in Figure 1, the remaining wound sizes in *IL36R^−/−^* mice were markedly smaller than those in *WT* mice at 24 hpw (4.09-fold). *IL36R* deficiency in DM mice resulted in accelerated wound healing with wound sizes 5.79-fold smaller than in diabetic WT mice.

### 3.6. IL-36R Deficiency Promotes IL-Ra Expression in DM Corneas

**Figure 6:**
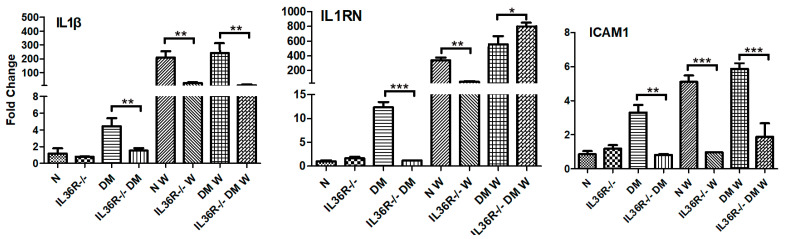
*IL-36R* deficiency promotes the expression of proinflammatory cytokines in NL and DM corneas. Corneal epithelial cells collected from NL and DM corneas during wounding and at 24 hpw were subjected to real-time PCR analyses. Each sample was normalized with actin as the internal control, and the results are expressed as the fold increase compared with the gene expression of epithelial cells from unwounded WT mouse corneas. *p* values were analyzed with a two-way ANOVA, followed by a Bonferroni test. *n* = 5, * *p* < 0.05, ** *p* < 0.01, *** *p* < 0.001. The results are representative of two independent experiments.

Our previous study revealed that the disturbance of IL-1β/IL-1Ra balanced expression is an important contributor to the pathogenesis of microbial keratitis and delayed epithelial wound healing in DM corneas [2,21,35]. To determine whether IL-36R signaling also modulates IL-1β and IL-1Ra expression, we assessed their expression levels in *WT* and *IL-36R^−/−^* mice with or without diabetes. In uninjured corneas of wild-type mice, hyperglycemia upregulated the expression of *IL-1β*, *IL-1Ra*, and *ICAM1*, whereas *IL-36R* deficiency attenuated this increase. While there was no litter effect detected in NL unwounded corneas, IL-36R deficiency significantly suppressed DM-associated elevated expression of *IL-1β*, *IL-1Ra*, as well as *ICAM1*, which may contribute to the low-grade inflammation found in DM corneas [36,37]. Their expression levels in healing epithelial cells were greatly increased in both NL and DM corneas. *IL-36R* deficiency repressed all three genes in NL but only repressed *IL-1β* and *ICAM* in DM corneas. *IL-36R* depletion resulted in the promotion of *IL-36Ra* expression in DM corneas (Figure 6).

### 3.7. Recombinant IL-1Ra and IL-36Ra Accelerated Delayed Epithelial Wound Healing in Diabetic B6 Mouse Corneas 

**Figure 7:**
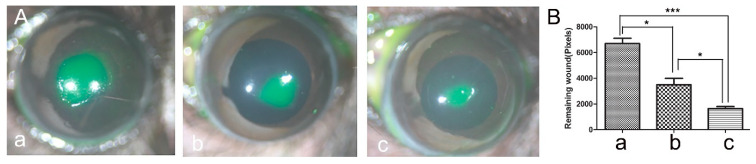
Effects of IL1Ra (anakinra) and recombinant mouse IL36Ra on epithelial wound healing in B6 mouse DM corneas. Mice were subconjunctivally injected with either 150 ng anakinra (**Ab**), recombinant mouse IL36Ra (**Ac**), or BSA as the control (**Aa**). A 2 mm epithelial debridement wound was made in each DM cornea, and wounds were allowed to heal for 24 h in vivo. The remaining wounds were photographed (10×) (**A**), and wound sizes were measured using Image J 1.47 (**B**). The results are presented as the mean ± SD. *p* values were analyzed with a one-way ANOVA, followed by a Bonferroni test. *n* = 5, * *p* < 0.05, *** *p* < 0.001. The results are representative of two independent experiments.

We recently reported that IL-1Ra and IL-36Ra had opposing effects on innate immunity against Pseudomonas aeruginosa infection [21]. Our previous study showed that IL-36R deficiency promotes IL-1Ra expression. To investigate this further, we treated DM mice with recombinant mouse IL-36Ra and human IL-1Ra (anakinra) before epithelial wounding. We found that both IL-1Ra and Il-36Ra significantly accelerated epithelial wound closure in DM mice, with Il-36Ra being more potent than IL-1Ra in DM corneas Figure 7).

## 4. Discussion

In this study, we investigated the role of IL-36R signaling in corneal epithelial wound healing in B6 mice. We demonstrated that IL-36R deficiency results in the acceleration of epithelial wound closure in both NL and DM corneas and that IL-36α and IL-36γ partially contribute to the inhibitory effects of IL-36R signaling on wound healing. Wounding induced the upregulation of the proinflammatory cytokines CXCL1 and CXCL2, while the expression of the AMPs S100A8 and S100-A9 was augmented in IL-36R^−/−^ corneas. Interestingly, LCN2 depletion also delayed epithelial wound closure, suggesting it is a downstream regulator of epithelial migration and proliferation. To further explore the role of IL-36R, WT and IL-36R^−/−^ mice were induced to develop type 1 diabetes. In WT mice, hyperglycemia profoundly affected epithelial wound closure; its effects on wound healing in IL-36R KO mice were less apparent. IL-36R deficiency suppressed wound-induced proinflammatory IL-1β and ICAM1 expression but promoted IL-1Ra expression in DM corneas. Functionally, exogenous IL-36Ra and IL-36Ra facilitated DM wound healing, with IL-36Ra being more effective. Taken together, we conclude that IL-36R signaling mediates corneal wound healing through its effects on gene expression, and targeting IL-36R signaling may ameliorate hyperglycemia-impaired tissue regeneration and repair.

Our previous study showed that IL-36 cytokines have basal expression in corneas, particularly epithelial cells, unlike members of the IL-1 cytokine subfamily [21,35]. While IL-36β is expressed in monocytes, B cells, neurons, and glia, IL-36α is mostly expressed in skin adaptive cells and upregulated in injured kidneys associated with the development of renal pathologies as well as hepatocellular carcinoma and some inflammatory/immune diseases [38,39]. IL-36γ is expressed in neutrophils, keratinocytes, and bronchial epithelial cells. Keratinocytes can stimulate IL-36R-expressing cells, such as dendritic cells and monocytes [40,41]. A study showed that skin injury increased the expression of IL-36γ in epidermal keratinocytes surrounding the wound edges to promote wound healing [7]. IL-36 cytokines are highly expressed in hyperproliferative keratinocytes and play an important role in the pathogenesis of skin diseases, such as psoriasis [4,42]. IL-36Ra is a receptor antagonist that inhibits the activation of IL-36R signaling, and its deficiency causes Generalized Pustular Psoriasis [42]. IL-36Ra and IL-1Ra have 52% homologous amino acid sequences and both function as receptor antagonists [39]. Interestingly, an infant with IL-36Ra deficiency was successfully treated with IL-1Ra anakinra [43]. Our study showed that two IL-36R agonists, IL-36α and 36γ, were upregulated, whereas IL-36Ra was downregulated, suggesting elevated signaling of IL-36R in healing or migratory corneal epithelial cells. 

The upregulation of IL-36R signaling in response to wounding suggests a supportive role in corneal epithelial wound healing. However, we observed that there were detectable increases in the rate of epithelial wound closure in IL-36α^−/−^, IL-36γ^−/−^ mice, although these were not significant. Remarkably, the depletion of IL-36R greatly accelerated epithelial wound closure. Hence, Il-36α and Il-36γ may have overlapping functions in response to wounding and/or suppressed epithelial wound healing in the cornea. IL-36α was reported to be upregulated in keratinocytes following mechanical wounding and was shown to play an important role in keratinocyte migration [44]. IL-36γ, through the induction of REG3A, results in keratinocyte proliferation and differentiation, thus promoting wound re-epithelialization and wound healing in the skin [7]. On the other hand, IL-36Ra deficiency was reported to delay full-thickness excisional wound healing, indicating that elevated IL-36R signaling dampens wound healing in B6 mouse skin [9]. The reason for the discrepancy in the role of IL-36R signaling in skin wound healing is unclear. Our results showing that IL-36R deficiency accelerates corneal epithelial wound healing are consistent with the IL-36Ra deficiency study of the skin [9]. 

How might IL-36R signaling modulate epithelial wound healing in the cornea? In diabetic mice, the downregulation of LCN2 was reported to facilitate macrophage polarization toward the M2 phenotype and improve impaired wound healing. Moreover, LCN2 deficiency significantly reduced gliosis, the recruitment of macrophages, and the production of inflammatory cytokines in diabetic mice, suggesting a critical role of LCN2 in the pathogenesis of diabetic encephalopathy [45]. On the other hand, downregulation of LCN2 was shown to significantly inhibit cell migration, invasion, angiopoiesis, and pyroptosis regulated by caspase-1, thus attenuating the progression of diabetic retinopathy [46]. Our results indicate a negative effect of wound-induced expression of LCN2 on corneal epithelial wound healing. Further enhancement of the expression of LCN2 may contribute to impaired wound healing in IL-36R-deficient mouse corneas. Whether macrophage phototypes are involved in the IL-36R–LCN2 axis or impaired epithelial wound closure remains to be determined.

The expression of LCN2 at the protein level was assessed, and the results on whether hyperglycemia augmented LCN2 expression were inconsistent, a limitation of our study. This inconsistency may be related to the fact that LCN2 is an extracellular protein that is released into the extracellular space by various cells, including epithelial cells, neutrophils, and macrophages [47]. Human keratinocytes were reported to be potent sources of chemokines following the exposure of IL-36 cytokines, leading to the recruitment of macrophages, T cells, and neutrophils [48]. Lipocalin-2 (LCN2), also known as neutrophil gelatinase-associated lipocalin (NGAL), is released by innate immune cells and can serve as an attractive biomarker of inflammation, ischemia, infection, and kidney damage. Consistent with our findings, the neutralization of LCN2 has been shown to control neutrophilic inflammation in experimental disease models of psoriasis, cardiovascular disease, alcoholic steatohepatitis, and nonalcoholic steatohepatitis [49]. IL-36R deficiency significantly enhanced corneal wound healing in type 1 diabetic mice and appeared to be more effective for promoting epithelial wound closure in diabetic corneas than in normal corneas. It is important to note that there are differences between type 1 and type 2 DM in the context of corneal wound healing and diseases. Delayed corneal wound healing is more commonly associated with Type 1 DM [22], and patients with Type 1 DM have a higher risk of developing corneal complications such as diabetic keratopathy, recurrent corneal erosions, and neurotrophic keratitis [50]. We used type 1 DM mice in this study. Mechanistically, we assessed the effects of IL-36R signaling on the expression of IL-1 cytokines in wild-type and IL-36R-deficient mice. We observed that, in diabetic corneas, IL-36R depletion resulted in enhanced expression of IL-1Ra but not IL-1β. In many tissues, IL-1β and IL-1Ra are paired to control inflammation [51]. Our previous studies showed that increasing IL-1Ra expression and/or the IL-1Ra/IL-1β balance promotes epithelial wound healing and innate corneal defense against Candida albicans and Pa Keratitis [35,52]. This study provides further support for the positive role of IL-1Ra in promoting epithelial wound healing, particularly in diabetic corneas. Importantly, the wound-induced upregulation of IL-1Ra but not IL-1β in diabetic corneas was further augmented by the inactivation of IL-36R signaling. 

## 5. Conclusions

The finding that IL-36R deficiency enhances IL-1Ra expression and promotes effective epithelial wound closure in DM corneas suggests the therapeutic potential of IL-1Ra and IL-1ra. Indeed, we observed that both IL-1Ra and IL-36Ra accelerated delayed corneal epithelial wound healing in DM mice. While human IL-1Ra anakinra has been used to control inflammation and symptoms in many human diseases, including rheumatoid arthritis and neonatal-onset multisystem inflammatory disease [53], the clinical use of IL-36Ra has not been fully explored [41,54]. Our study provides further evidence that IL-36Ra is a therapeutic reagent for treating diabetic wound healing, as well as psoriasis [54,55], asthma [41], and type 2 DM-associated obesity, insulin resistance, and inflammation [56].

## Data Availability

The data presented in this study are available on request from the corresponding author. The data are not publicly available due to privacy reasons.
